# A Study about Perceptions of Kimono among College Students and Kimono Enthusiasts: Is It Difficult to Move in a Kimono?

**DOI:** 10.12688/f1000research.149040.1

**Published:** 2024-04-18

**Authors:** Kozue Miyashiro, Kazuya Sasaki, Tomoharu Ishikawa, Hiroshi Mori

**Affiliations:** 1 Cooperative Faculty of Education, Utsunomiya University, 350 Mine Machi, Utsunomiya City, Tochigi Prefecture, 321-8505, Japan; 2 Faculty of Engineering, Utsunomiya University, 7–1–2 Yoto, Utsunomiya City, Tochigi Prefecture, 321-8585, Japan

**Keywords:** Perceptions of kimono, kimono enthusiasts, kimono as daily wear, wearing experience, challenges when wearing a kimono, questionnaire

## Abstract

**Background:**

Kimono is being reevaluated for its sustainability aspects, such as having fewer offcuts in the production process due to its structural differences from Western-style clothes and its high reusability due to the adaptability to individuals’ body shapes. On the other hand, once a common attire for daily wear in Japan, kimono has transitioned to being worn only on special events and the kimono-related industry has also shrunk. To stimulate demand for kimono, it is essential to familiarize younger generations with its potential as daily wear.

**Methods:**

A questionnaire survey on perceptions of kimono was conducted among two groups in Japan: 211 college students and 50 kimono enthusiasts. The questionnaire included demographic questions and psychometric scales, primarily focusing on their kimono experiences, challenges associated with wearing kimono, their perceptions of kimono and Western-style clothes, and their attitudes towards kimono.

**Results:**

The results revealed that a majority of students had worn kimono before, though they found it difficult to move while wearing it. In contrast, kimono enthusiasts evaluated it as easier to move, hard to become disheveled, and casual. They also rated the ease of wearing Western-style clothes lower compared to students, and this tendency intensified with the length of enthusiast experience. Furthermore, the findings indicated that enthusiasts regarded the kimono more as daily wear compared to students, while still deriving enjoyment from it as formal attire in special events.

**Conclusions:**

These results suggest that the cognition that Western-style clothes are easy to move and kimono is not may change with experiences. Therefore, providing opportunities for people in Japan to acquire how to wear kimono in comfortable ways possibly impacts their perceptions of kimono.

## 1. Introduction

### 1.1 The decline of kimono wearing in Japan and its background

*Kimono*, a traditional Japanese attire, is being reevaluated for its sustainability aspects, such as having fewer offcuts in the production process due to its structural differences from Western-style clothes and its high reusability due to the adaptability to individuals’ body shapes. In Japan's school education system, there is also a focus on preserving traditional culture. The current Curriculum Guidelines for junior high schools issued in Heisei 29 ( Ministry of Education in Japan, 2017) mention the possibility of introducing traditional Japanese clothes and teaching the basics of wearing them.

On the other hand, kimono is worn less frequently as daily wear in these days. According to a kimono consumer survey ( Ministry of Economy, Trade and Industry in Japan, 2015), targeting women aged 20 and over, 76.7% of respondents identified “ceremonial occasions” as situations in which they want to wear kimono in the future. In light of the current situation where there are limited opportunities to wear kimono, Yoshida (2014) claimed that kimono is no longer a fashion worn even in formal settings, but rather positioned as a special costume worn only during specific life events.

The kimono-related industry has also shrunk, once estimated as much as 2 trillion-yen industry, have fell to approximately 300 billion yen in 2011 ( Yoshida, 2014). In terms of the preservation of Japan's traditional heritage, the kimono is currently facing a crisis.

Adachi (2015) discusses the expansion of kimono distancing in modern clothes culture. The catalyst was the rapid proliferation of Western-style lifestyles, leading to a decline in demand for kimono. To compensate for the decrease in kimono sales, the industry pursued a strategy of developing only high-priced formal kimono, neglecting the casual kimono that was once worn in daily life. Consequently, the market became saturated with formal kimono, while casual kimono disappeared. The kimono, which used to be daily wear, became increasingly unaffordable for the average consumer and limited in occasions suitable for wearing. In order to encourage consumers to purchase these products, the industry emphasized their “year-round wearability” and “timelessness” as selling points. However, as a result, seasonal relevance diminished from the kimono displayed in stores, and there has been an increase in conservative classical patterns over more creatively expressive designs.

### 1.2 Current study

The kimono, by its nature, can be both casual and formal, depending on its type and how it is worn. Awareness of this versatility is crucial in recognition of the allure of the kimono. To stimulate demand for kimono, it is necessary to examine how individuals form their impressions of kimonos based on personal experiences. Particularly for women, the comfort of wearing a kimono can vary greatly depending on the position and width of the *obi* (sash) and the tightness of its tie. If one's past experiences with kimono have been limited to formal occasions, the perception of kimono may become fixed as rigid and cumbersome attire.

Therefore, this study aimed to first investigate the experiences and perceptions regarding kimono among younger generations who may not be familiar with them. Conversely, it explored what aspects of kimono appeal to individuals who are already familiar with them, and how these perceptions differ from those of the younger generation. The study targeted university students (both undergraduate and graduate) as well as kimono enthusiasts (individuals attending kimono classes). By comparing data from these groups, the study also examined the backgrounds of individuals who became kimono enthusiasts, proposing initiatives to stimulate demand for kimono, particularly among the younger generation.

To understand individuals' involvement with kimono, we utilized questionnaire items concerning their experiences of wearing kimono and the associated challenges, perceptions of kimono and Western-style clothes ( Mino & Niwa, 2008), and inquiries into their attitudes towards kimono ( Yoshida, 2014).

## 2. Methods

### 2.1 Consent and ethical considerations

This study was conducted after obtaining approval from the Research Ethics Committee at Utsunomiya University for research involving human subjects. The survey targeting students was approved on January 12, 2023 (Approval Number H22-0115), and a revised version including enthusiasts as subjects was subsequently approved on March 8, 2023 (Approval Number H22C-0115). The entire study was conducted in adherence with the Declaration of Helsinki ( World Medical Association, 2013).

The survey, titled “survey on kimono among college students” and “survey on kimono among kimono enthusiasts”, respectively, involved distributing paper questionnaires during break times on campus or at kimono classes in the Kanto region. The detailed study hypothesis was not explained in advance to avoid biasing effects on the results.

It was emphasized that participation was voluntary, and participants were assured that their decision to participate or not would not result in any disadvantages, the survey responses would be used for research purposes only, all responses would be treated anonymously and analyzed statistically, and the survey data would be securely stored. Participants were also informed that they could withdraw from the survey at any time without facing any repercussions, even after agreeing to participate. They were instructed to proceed with the survey only if they agreed to these conditions.

### 2.2 Participants

A total of 211 university and graduate students from a university in Japan participated in the survey (79 males, 128 females; 2 respondents chose “no response” and 2 did not select any option; mean age 19.20 ± 1.85 years; 200 Japanese nationals, 2 Mongolian nationals, 9 Chinese nationals). Additionally, 50 individuals attending kimono classes in the Kanto region, participated (4 males, 36 females; mean age 63.00 ± 14.48 years, range 23-80 years, 2 respondents did not provide their age; mean duration of kimono enthusiast 17.42 ± 14.76 years, range 1-60 years, 2 respondents did not provide their duration of being kimono enthusiasts).

### 2.3 Data collection instrument

The questionnaire consisted of demographic questions and psychometric scales.

(1) **Demographic questions:** In the survey targeting students, this section consisted of items asking for gender, age, and nationality. For international students, respondents were also asked about the number of years since they arrived in Japan. In the survey targeting kimono enthusiasts, participants were asked to provide information on gender, age, current occupation, and the duration of their experience as a kimono enthusiast (in years).
(2) **Experience with kimono:** This section utilized items based on Mino & Niwa (2008; https://www.jstage.jst.go.jp/article/senshoshi1960/49/11/49_11_793/_article/-char/ja/). Participants were presented with ten scenarios with a high likelihood of wearing kimono (e.g., summer festivals, Shichi-Go-San; a traditional Japanese celebration for children who are 3/5/7 years old, Coming of Age ceremonies) as well as an “Other” option, allowing them to select all scenarios in which they had worn kimono. If they chose “Other,” they were provided with a space to specify the specific scenario.
(3) **Challenges of wearing kimono:** This section utilized items based on Mino & Niwa (2008). Participants were presented with ten options that could be considered challenges while wearing kimono (e.g., difficult to move, hot, cold), along with an “others” option allowing them to specify additional challenges if applicable. Participants were instructed to select all options that applied to them. If they chose “others,” they were provided with a space to specify the specific challenges.
(4) **Perceptions of kimono and Western-style clothes:** This section utilized items from Mino & Niwa (2008), focusing on the perception of clothes such as Western-style clothes, *furisode* (long-sleeved kimono for young women), and *yukata* (casual summer kimono). Participants were instructed to mark their perception on a scale of 0 to 10 for each of 11 adjective pairs (e.g., formal—casual) presented along a continuum. After completing the survey on kimono, participants were instructed to respond to the same 11 adjective pairs regarding Western-style clothes.
(5) **Attitudes towards kimono:** Participants were asked to respond to 38 items regarding attitudes towards kimono, based on Yoshida (2014; https://www.consortium.or.jp/seisaku/5137). Examples of these items include “I want to wear kimono as casual wear in my daily life.” and “Wearing a kimono turns my mood off.” Responses were solicited using a 7-point scale ranging from “1: Not at all applicable” to “7: Very applicable.”

## 3. Results and Discussion

### 3.1 Past experience of wearing kimono

3.1.1 Experience and occasions of wearing kimono

Regarding students' experiences of wearing kimono, 36 participants (17.06% of the participants) reported that they had never worn kimono. Among them, 9 were female students (7.03% of female students), and 27 were male students (34.18% of male students). It is evident that even among the younger generation of students, the majority have had opportunities to wear kimono. In the case of kimono enthusiasts, all 50 participants reported having experience wearing kimono.

**Figure 1.**
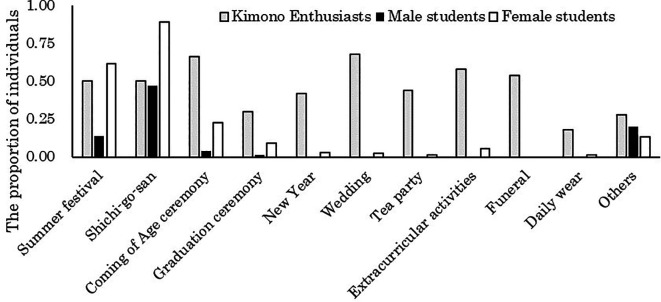
Occasions where participants wore kimonos in the past (multiple responses possible).

Figure 1 presents the aggregation of occasions for wearing kimono when considering the number of individuals who have worn kimono as 100% for male students, female students, and kimono enthusiasts, respectively.

3.1.2 Challenges of wearing kimono

**Figure 2.**
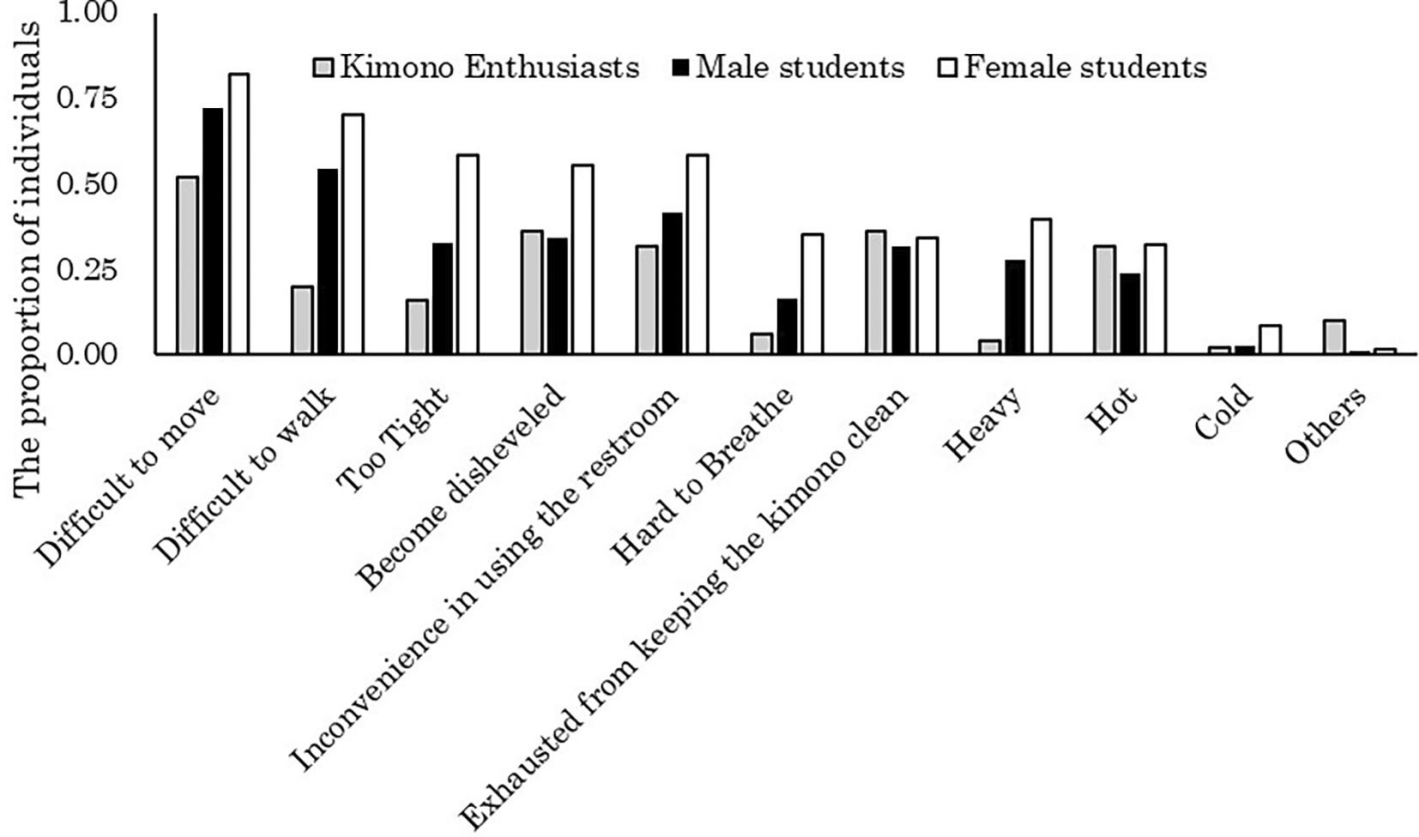
Challenges of wearing kimono (multiple responses possible).

The aggregated results of the challenges encountered when wearing kimono for both students and kimono enthusiasts are illustrated in Figure 2. The majority of students (72.2% of males and 82.0% of females) reported experiencing difficulty in movement while wearing kimono. Conversely, among kimono enthusiasts, it is evident that a relatively smaller proportion (52.0%) did so.

### 3.2 Differences in perceptions of kimono

We conducted an exploratory factor analysis on the overall data (students and kimono enthusiasts) regarding the image of kimono. In each item, missing values ranged from one to six participants and they were handled using list-wise deletion. As a result, only one factor was extracted. Two items, “Ease of aligning with fashion trends—Difficulty in alignment with fashion trends” (-.170) and “Fashionable—Not Fashionable” (.084), with factor loadings below .4 were omitted from subsequent analysis.

The remaining nine items ( Table 1) were averaged to calculate the composite score for perceptions of kimono. Basic statistics for each group are presented in Table 2.

**Table 1.** Results of factor analysis on scores for perceptions of kimono.

Items	Factor 1	Communality
Difficult to move—Easy to move	**.844**	.712
Hard to wear—Easy to wear	**.810**	.656
Tight—Loose	**.756**	.572
Heavy—Light	**.714**	.509
Easy to became disheveled—Hard to became disheveled	**.698**	.487
Traditional—Modern	**.584**	.341
Formal—Casual	**.573**	.329
Expensive—Affordable	**.567**	.321
Conspicuous—Not conspicuous	**.479**	.229

**Table 2.** Basic statistics of the composite score for perceptions of kimono.

	*M*		*SD*
All participants	2.05		1.98
Students	1.68		1.68
Kimono Enthusiasts	3.62		2.38

**Figure 3.**
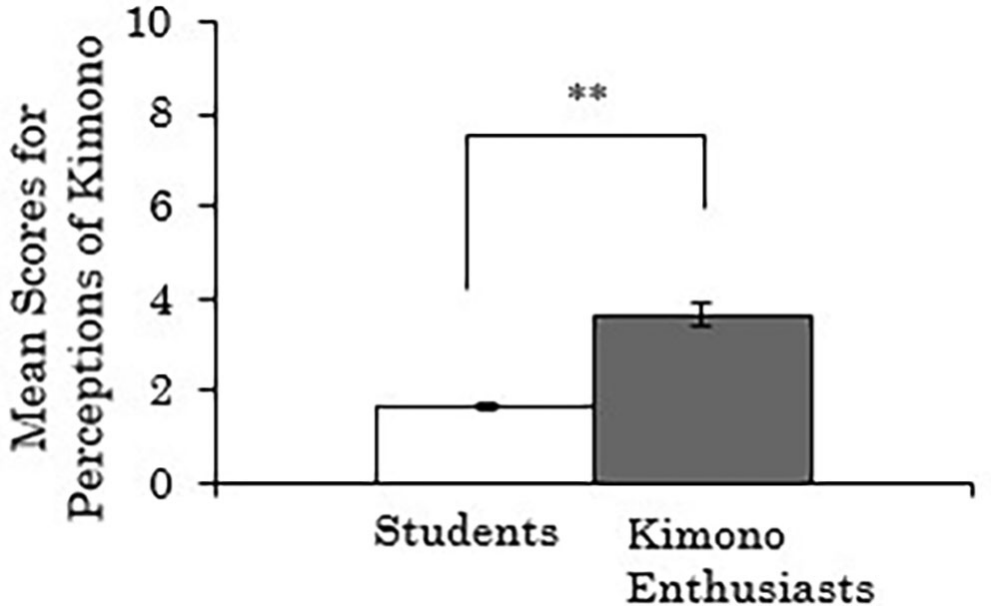
Group comparison of scores for perceptions of kimono (the error bars represent standard errors).

We conducted independent samples t-tests to examine whether there were differences in the composite scores for perceptions of kimono based on participants' attributes (students vs. kimono enthusiasts). Missing values were handled using pair-wise deletion. The results revealed a significant difference between groups ( *t*(258)=-10.314, *p*<.001), indicating that the kimono enthusiasts rated kimonos as more comfortable to wear, less prone to coming disheveled, and more casual compared to the students ( Figure 3).

Additionally, to explore whether this tendency among kimono enthusiasts was strengthened with a longer duration of enthusiasm, we conducted a correlation analysis between the duration of kimono enthusiasm and the composite score for perceptions of kimono, focusing solely on data from kimono enthusiasts. However, no significant correlation was found ( *r*=.185, *p*=.213).

### 3.3 Differences in perceptions of Western-style clothes

We also conducted an exploratory factor analysis on the scores for perception of Western-style clothes, encompassing all available data. In each item, missing values ranged from one to three participants and they were handled using list-wise deletion. As a result, three factors were extracted. Based on the content of each questionnaire items, we labeled the first factor as “easy to move,” the second factor as “easy to wear in daily life,” and the third factor as “fashionable.” One item, “conspicuous—not conspicuous” (.368), with a factor loading below.4, was omitted from subsequent analysis. The remaining 10 items ( Table 3) were averaged to calculate the composite score for perceptions of Western-style clothes.

**Table 3.** Results of factor analysis on score for perceptions of Western-style clothes.

Items	Easy to move	Easy to wear in everyday life	Fashionable	Communality
Easy to became disheveled—Hard to became disheveled	**.814**	-.138	.035	.526
Hard to wear—Easy to wear	**.796**	-.026	-.094	.647
Heavy—Light	**.706**	.075	.043	.561
Traditional—Modern	**.694**	-.040	-.076	.474
Difficult to move—Easy to move	**.483**	.380	.004	.614
Expensive—Affordable	-.098	**.808**	-.035	.567
Formal—Casual	-.067	**.726**	-.078	.485
Tight—Loose	.166	**.657**	.083	.588
Easy to wear in line with fashion trendsl—Difficult to wear in line with fashion trends	.016	.004	**.868**	.747
Fashionable—Not Fashionable	-.038	-.052	**.587**	.369

To note, for the “fashionable” score for Western-style clothes, we reversed the numerical values to indicate higher scores representing “more fashionable” before calculating the mean. Basic statistics for each group are presented in Table 4.

**Table 4.** Basic statistics of each composite score for perceptions of Western-style clothes.

Factors		*M*	*SD*
Easy to move	All participants	8.44	7.43
	Students	8.49	7.57
	Kimono Enthusiasts	8.21	6.82
Easy to wear in daily life	All participants	3.21	1.72
	Students	2.89	1.69
	Kimono Enthusiasts	4.59	1.84
Fashionable	All participants	1.88	3.05
	Students	1.83	3.02
	Kimono Enthusiasts	1.97	2.76

We conducted independent t-tests below to examine differences between the groups (students vs. kimono enthusiasts) on each score. Missing values were handled using pair-wise deletion.

3.3.1 Score for “easy to move”

First, there was no significant difference in score for “easy to move” between the groups ( *t*(257)=1.299, *p*=.195).

However, a correlation analysis between the duration of being kimono enthusiasts and the score for “easy to move” in Western-style clothes using data only from the kimono enthusiasts revealed a weak but significant negative correlation ( *r*=-.253, *p*=.090). This suggests that while the evaluation of “easy to move” in Western-style clothes does not differ significantly between the students and kimono enthusiasts overall, those with longer experience in kimono appreciation tend not to perceive Western-style clothes as particularly easy to move. As there is a positive correlation between the duration of being kimono enthusiasts and age ( *r*=.419, *p*=.004), one interpretation of the above results alone could be that ease of movement in daily life decreases with age. However, as mentioned earlier, no correlation was found between the scores for perceptions of kimono (which also include the evaluation of ease of movement in kimono) and the duration of being kimono enthusiasts. Therefore, it would be more appropriate to interpret that the longer the duration of experience as a kimono enthusiast, the lower the evaluation of ease of movement in Western-style clothes.

3.3.2 Score for “easy to wear in daily life”

**Figure 4.**
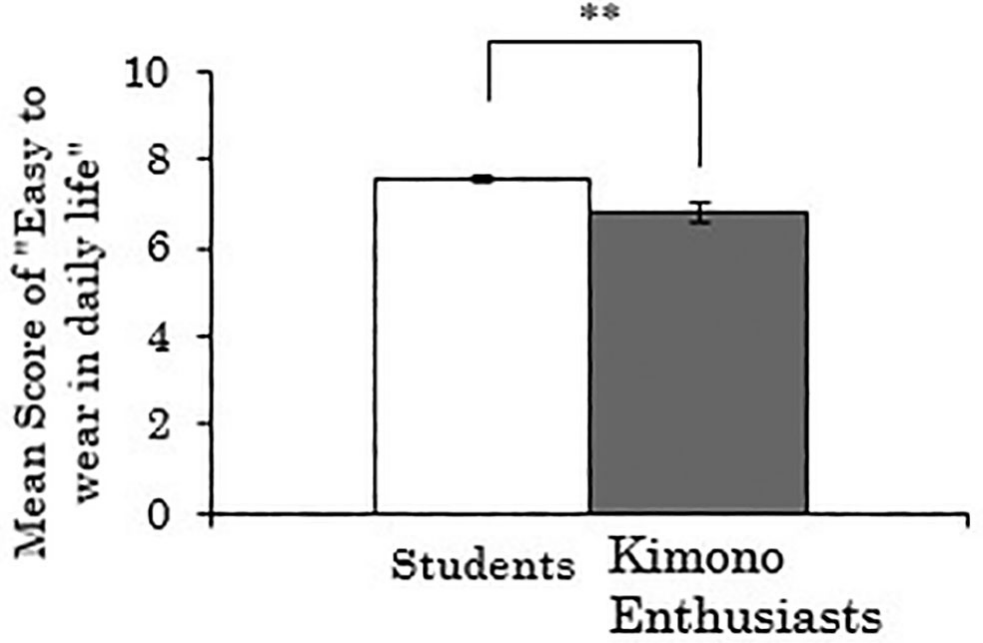
Group comparison of scores for “easy to wear in daily life” of Western-style clothes (the error bars represent standard errors).

Next, when the dependent variable was the score for “easy to wear in daily life,” there was a significant difference between the groups ( *t*(258)=3.174, *p*=.002), indicating that the kimono enthusiasts rated the ease of wearing Western-style clothes lower than the students (see Figure 4). Furthermore, a correlation analysis was conducted using data only from the kimono enthusiasts to examine the relationship between the duration of being kimono enthusiasts and the score for “easy to wear in daily life” of Western-style clothes. The results showed a weak but significant negative correlation ( *r*=-.264, *p*=.073). Thus, there is a possibility that the tendency observed in the t-test mentioned above, where the kimono enthusiasts rated the ease of wearing Western-style clothes lower, becomes stronger with a longer duration of kimono enthusiasm.

3.3.3 Score for “fashionable”

**Figure 5.**
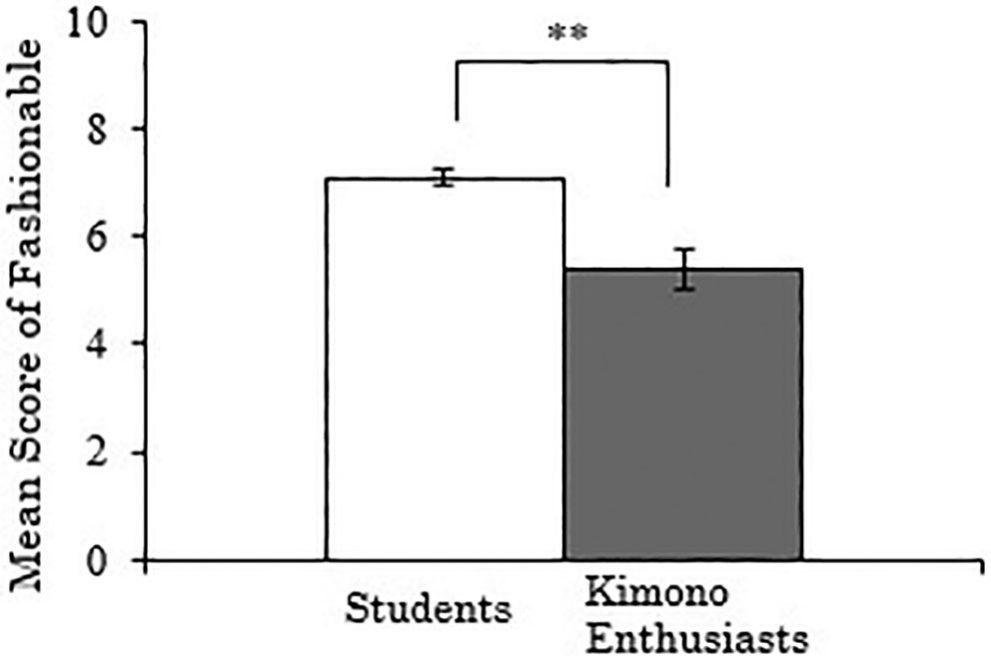
Group comparison of scores for “fashionable” of Western-style clothes (the error bars represent standard errors).

The results, with the “fashionable” score for Western-style clothes as the dependent variable, showed a significant difference between the groups ( *t*(257)=-4.147, *p*<.001), indicating that the students rated the fashionability of Western-style clothes higher than the kimono enthusiasts ( Figure 5). A correlation analysis was conducted using data only from the kimono enthusiasts, examining the relationship between their duration of ”kimono enthusiasm and the “fashionable” score for Western-style clothes, but no significant correlation was found ( *r*=-.136, *p*=.369).

### 3.4 Differences in attitudes towards kimono

Next, we conducted an exploratory factor analysis on attitudes towards kimono using the entire dataset (students and kimono enthusiasts). In each item, missing values ranged from one to four participants and they were handled using list-wise deletion. As a result, three factors were extracted. Based on the content of each questionnaire item, the first factor was labeled “kimono as daily wear,” the second factor “kimono as a special day wear,” and the third factor “adviser.” 18 items with factor loadings below. 4 were removed from subsequent analysis.

For the remaining 20 items (see Table 5), reverse scoring was applied to items as necessary to ensure alignment with the factor names, and composite scores were calculated by averaging scores within each factor. Basic statistics for each group are presented in Table 6.

**Table 5.** Factor analysis results for scores of attitudes towards kimono (* denotes reversed items).

Items	Kimono as daily wear	Kimono as a special day wear	Adviser	Communality
I want to wear kimono as casual wear in my daily life.	**.707**	.005	-.006	.504
I think coordinating kimono is more fun than western-style clothes.	**.649**	.068	-.095	.495
Kimono is home wear (daily wear).	**.614**	-.117	-.065	.399
I think it's okay to wear a kimono as I like.	**.564**	-.213	.156	.299
Kimono is a fashion for enjoying style.	**.529**	.218	.052	.342
I think coordinating western-style clothes is more fun than kimono.*	**-.519**	.078	.022	.271
I think kimonos look good in modern living spaces.	**.488**	.089	-.107	.312
Wearing a kimono turns my mood off.	**.480**	-.238	.040	.243
Kimono is also clothing, so I have to be able to put it on myself.	**.429**	.274	-.039	.313
I would like to purchase a kimono made with carefully selected threads and manufacturing methods even if it is expensive.	**.427**	.349	-.026	.362
I am fascinated by the fact that kimono is a handicraft that takes a lot of time and effort.	.214	**.652**	.076	.490
I am attracted to “graceful (はんなりとした)” kimonos like Kyoto-Yuzen.	-.088	**.643**	.008	.402
I am attracted to “stylish (粋な)” kimono like Edo-Komon.	.224	**.587**	-.028	.446
I am attracted to innovative colors and patterns that are not found in western style clothes.	.020	**.579**	.035	.333
Kimono is a traditional culture that should be preserved.	-.314	**.526**	-.053	.325
I think kimonos look good in traditional living spaces.	-.217	**.476**	.114	.253
I think the rules of kimono should be followed.	-.432	**.475**	-.070	.344
Wearing a kimono turns my mood on.	.001	**.412**	-.140	.210
I know people and shops where I can easily consult about kimonos.	.040	-.086	**-.958**	.924
I don't know anyone or shops I can easily talk to about kimono.*	-.047	.158	**.862**	.748

**Table 6.** Basic statistics of each composite scores for attitudes towards kimono.

Factors		*M*	*SD*
Kimono as daily wear	All participants	3.22	1.67
	Students	2.97	1.61
	Kimono Enthusiasts	4.30	1.45
Kimono as a special day wear	All participants	5.25	1.41
	Students	5.28	1.43
	Kimono Enthusiasts	5.13	1.32
Adviser	All participants	3.19	2.10
	Students	2.84	2.01
	Kimono Enthusiasts	4.66	1.76

We conducted independent t-tests below to compare the scores between the groups (students vs. kimono enthusiasts). Missing values were handled using pair-wise deletion.

3.4.1 Score for “kimono as daily wear”

**Figure 6.**
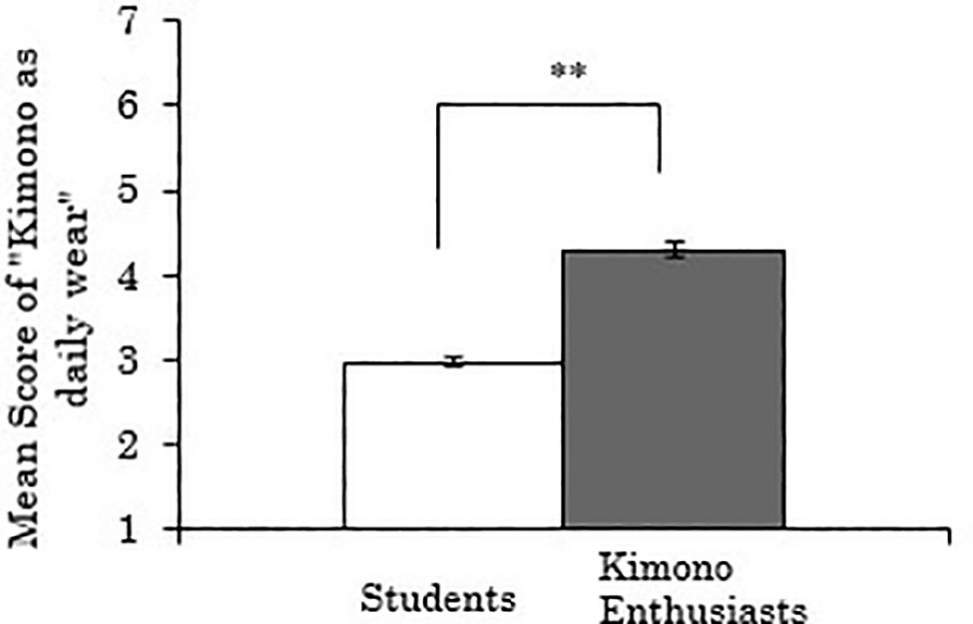
Group comparison of scores for “kimono as daily wear” (the error bars represent standard errors).

We first analyzed the score for “kimono as daily wear” as the dependent variable. The results showed a significant difference between the groups ( *t*(258)=-11.107, *p*<.001), indicating that kimono enthusiasts perceived kimono more as daily wear than students ( Figure 6). Using data only from the kimono enthusiasts, a correlation analysis between the duration of being kimono enthusiasts and the score for “kimono as daily wear” was also conducted, but no significant correlation was found ( *r*=.173, *p*=.241).

3.4.2 Score for “kimono as a special day wear”

Next, we analyzed the score for “kimono as a special day wear” as the dependent variable. There was no significant difference between the groups ( *t*(258)=1.186, *p*=.237). This suggests that while enthusiasts may view kimono as more of a daily wear compared to students, they do not necessarily dismiss the enjoyment of wearing it as special attire. It can be inferred from the results that the kimono enthusiasts perceive kimono as adaptable clothes that can serve both daily and formal purposes, depending on the type of kimono and how it is worn.

A correlation analysis was conducted between the duration of being kimono enthusiasts and the score for “kimono as a special day wear” using data exclusively from the kimono enthusiasts, but no significant correlation was observed ( *r*=.155, *p*=.293).

3.4.3 Score for “adviser”

**Figure 7.**
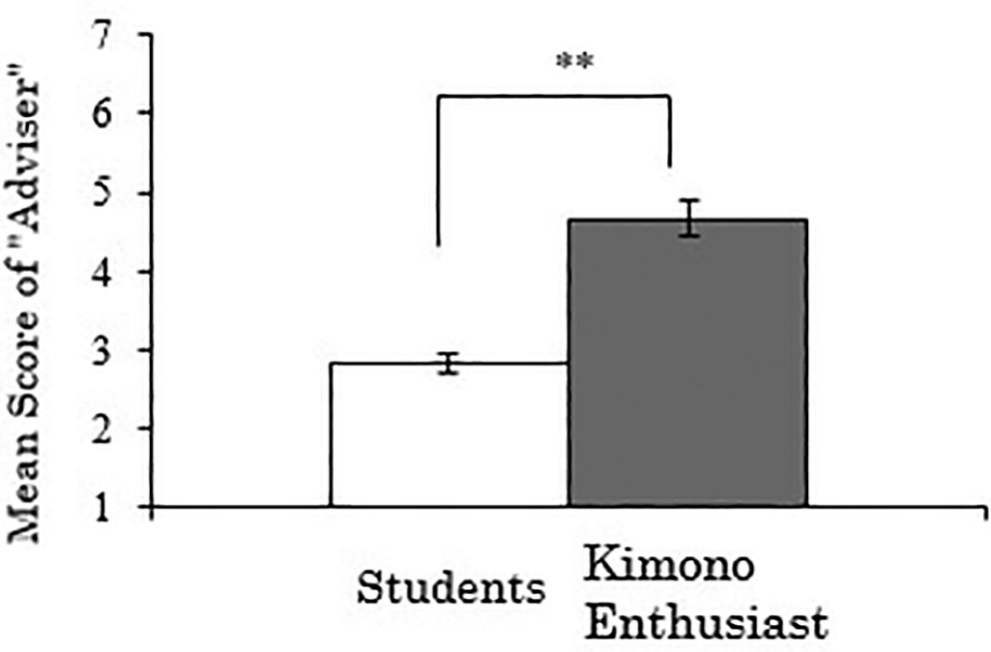
Group comparison of scores for “adviser” (the error bars represent standard errors).

We analyzed the “adviser” score as the dependent variable. As the results, there was a significant difference between the groups ( *t*(258)=-6.181, *p*<.001), indicating that kimono enthusiasts are more likely to have someone they can casually consult about kimono compared to students ( Figure 7). Whether this result reflects the need for advice on kimono among enthusiasts, who likely have more opportunities to wear kimono, or conversely, whether the availability of someone to consult about kimono has contributed to becoming an enthusiast, warrants further investigation.

A correlation analysis was conducted between the duration of kimono enthusiasm and the “adviser” score using data exclusively from the kimono enthusiasts, but no significant correlation was observed ( *r*=.074, *p*=.617).

## 4. Conclusion

In this study, a questionnaire survey was conducted with Japanese college students and kimono enthusiasts to investigate their experiences with wearing kimono and its challenges, their perceptions of kimono and Western-style clothes, and their attitudes towards kimono.

Firstly, the results indicated that a significant portion of students had opportunities to wear kimono in the past while many students experienced challenges such as difficulty in movement while wearing kimono. This could be attributed to the fact that students' experiences primarily involved wearing kimono for formal occasions or events such as Shichi-Go-San (a traditional rite of passage), where they may have tightly dressed in kimono to prevent them from coming undone. This suggests a consistent trend where students perceive Western-style clothes as more comfortable for movement compared to kimono, reflected across scores for perceptions of kimono, perceptions of Western-style clothes, and attitudes towards kimono. In other words, it seems that the versatility of kimono, which can be casual and easy to move in depending on the type and styling, is not recognized by the younger generation who have only experienced it as formal attire. Adachi (2015) observed that when considering the task of dressing a bedridden individual, it is apparent that kimonos are far more practical than Western-style clothes. Adachi argued that the notion that the popular belief as “Western-style clothes are functional while kimonos are not” stems from the perspective of able-bodied individuals, and negates the inherent flexibility and adaptability of traditional kimono attire.

On the other hand, it seems that among kimono enthusiasts, the tendency to perceive Western-style clothes as more functionally superior to kimono has been alleviated. The kimono enthusiasts rated kimonos as easier to move, harder to become disheveled, and more casual compared to students. They rated the ease of wearing Western-style clothes lower than college students, with this tendency becoming more pronounced among those with longer experience as enthusiasts. Also, there is a trend for the evaluation of the ease of movement in Western-style clothes to decrease as the duration of enthusiasm increases. Furthermore, while the kimono enthusiasts considered kimonos more of a “daily wear” compared to the college students, they did not deny the enjoyment of wearing them as formal attire.

From these results, it can be inferred that the tendency to perceive kimono as more functionally inferior to Western-style clothes may change as individuals accumulate experiences related to kimono. Conversely, to change the negative perceptions of kimono among young people, providing opportunities for them to experience more comfortable ways of wearing kimono could have an impact. Ensuring an adequate number of classes, support systems for dressing in coeducation environments would be key issues to be addressed, creating sufficient opportunities in Japanese school education.

Another distinction between the students and kimono enthusiasts is the availability of supportive environments for seeking advice about kimonos among the enthusiasts. This implies that newcomers, unfamiliar with kimono attire, could engage with the kimono community through these advisory channels and potentially develop into kimono enthusiasts. Further investigation through interviews will be needed to delve into this process of participation.

Currently, the inbound demand, which has been severely affected by the COVID-19 pandemic, is gradually recovering. Conducting surveys to understand the perceptions and needs of individuals with cultural backgrounds different from Japanese, regarding kimono, will become increasingly important. From the perspective of Sustainable Development Goals (SDGs), there is also a possibility of growing attention towards kimono as sustainable clothing. Therefore, it is necessary to conduct surveys targeting foreign tourists to Japan and overseas kimono enthusiasts, and comparing the results with those in Japan. The potential confounding between years of kimono enthusiasm and age also needs to be carefully discussed in future studies. By accumulating such insights, we aim to contribute to the expansion of kimono demand in the future.

## Consent and ethical considerations

This study was conducted after obtaining approval from the Research Ethics Committee at Utsunomiya University for research involving human subjects. The survey, titled “survey on kimono among college students” (approved on January 12, 2023; Approval Number is H22-0115) and “survey on kimono among kimono enthusiasts” (approved on March 8, 2023; Approval Number H22C-0115), respectively, involved distributing paper questionnaires during break times on campus or at kimono classes in Kanto region. The survey targeting students was conducted from January to June 2023, while the survey targeting enthusiasts was conducted from April to May 2023. All participants were confirmed to be adults in Japan at the time of participation. The entire study was conducted in adherence with the Declaration of Helsinki.

## Data Availability

Utsunomiya University Academic Institutional Repository: Is It Difficult to Move in a Kimono? A Study about Perceptions of Kimono among College Students and Kimono Enthusiasts. https://doi.org/10.24565/0002000161 ( Miyashiro *et al.*, 2024). This project includes the following underlying data:
• Kimono_survey.xlsx (Responses from participants used in this study) Utsunomiya University Academic Institutional Repository: Is It Difficult to Move in a Kimono? A Study about Perceptions of Kimono among College Students and Kimono Enthusiasts. https://doi.org/10.24565/0002000161 ( Miyashiro *et al.*, 2024). This project contains the following extended data:
• Kimono Questionnaire for Enthusiasts (A complete questionnaire for kimono enthusiasts translated into English) • Kimono Questionnaire for Students (A complete questionnaire for students translated into English) •
STROBE_checklist_cross-sectional-F1000.docx (A reporting checklist according to STROBE cross-sectional research guidelines) • Flow chart.pptx (Diagram showing the relationship between research content and procedures) The data is available under the terms of the Creative Commons Zero “No Rights Reserved” data waiver (CC0 1.0 Public Domain Dedication).
