# Supplementary material for: A Study about Perceptions of Kimono among College Students and Kimono Enthusiasts: Is It Difficult to Move in a Kimono?
Source: F1000Res. 2024 Sep 20;13:287. Originally published 2024 Apr 18. [Version 3] doi: 10.12688/f1000research.149040.3 (PMC11211731; doi:10.12688/f1000research.149040.3)
Supplement: Peer review history

# Reviewer report: Reviewer response for version 3

**DOI:** 10.5256/f1000research.172112.r325512

**Published:** 2024-09-23

**Authors:** Lasni Buddhibhashika Jayasooriya

**Affiliations:** 1 Department of Education, Kyushu University, Fukuoka, Japan

No further comments to make

Is the work clearly and accurately presented and does it cite the current literature?

Partly

If applicable, is the statistical analysis and its interpretation appropriate?

I cannot comment. A qualified statistician is required.

Are all the source data underlying the results available to ensure full reproducibility?

Yes

Is the study design appropriate and is the work technically sound?

Yes

Are the conclusions drawn adequately supported by the results?

Partly

Are sufficient details of methods and analysis provided to allow replication by others?

Yes

Reviewer Expertise:

Female schoolteachers' dress code, society and culture, school curriculum

I confirm that I have read this submission and believe that I have an appropriate level of expertise to confirm that it is of an acceptable scientific standard.

# Reviewer report: Reviewer response for version 2

**DOI:** 10.5256/f1000research.168270.r296344

**Published:** 2024-07-09

**Authors:** Ellen McKinney

**Affiliations:** 1 The University of Alabama, Tuscaloosa, Alabama, USA

I have no further comments to make.

Is the work clearly and accurately presented and does it cite the current literature?

Partly

If applicable, is the statistical analysis and its interpretation appropriate?

I cannot comment. A qualified statistician is required.

Are all the source data underlying the results available to ensure full reproducibility?

Yes

Is the study design appropriate and is the work technically sound?

Yes

Are the conclusions drawn adequately supported by the results?

Yes

Are sufficient details of methods and analysis provided to allow replication by others?

Yes

Reviewer Expertise:

Japanese fashion, Kimono, Zero-waste apparel design

I confirm that I have read this submission and believe that I have an appropriate level of expertise to confirm that it is of an acceptable scientific standard.

# Reviewer report: Reviewer response for version 1

**DOI:** 10.5256/f1000research.163443.r293693

**Published:** 2024-06-27

**Authors:** Lasni Buddhibhashika Jayasooriya

**Affiliations:** 1 Department of Education, Kyushu University, Fukuoka, Japan

The research adopts a quantitative approach to explore the experiences, challenges, perceptions, and attitudes towards wearing kimono among college students and kimono enthusiasts. The methodology employed is clearly articulated, and the paper presents the data and results appropriately. However, I find my statistical knowledge insufficient to provide detailed comments on the chosen statistical tests and their usage.

 The authors discuss literature, highlighting the decline in kimono wearing. It would have been more informative if the distinction between wearing kimono in "daily life" and at work had been elaborated. Before World War II, it was common for schoolteachers to wear kimono, but this practice shifted to Western-style attire. While Westernization significantly contributed to this shift, other factors such as economic considerations, safety, and productivity-related issues also played a role (See T, Namba,2008 [Ref 1]] and UGLB, Jayasooriya, et. al., 2020 [Ref 2]).

 There is a considerable difference between the mean age of the college student group and that of kimono enthusiasts, which may influence their daily activities and preferences for "daily wear". Additionally, age differences might affect exposure to cultural values and knowledge, resulting in varying attitudes towards kimono. These showcase limitations of comparison between the two groups, which would be appropriate to mention. On the other hand, the perceptions of kimono enthusiasts are biased in favor of kimono. Therefore, in the conclusion section of the abstract, it would be appropriate to specify that 'the ease of movement in Western clothing is alleviated as the experience of wearing kimono increases' is a perception that may not be shared by college students since that finding is based on kimono enthusiasts' data. Moreover, understanding what inspires kimono enthusiasts to embrace kimono culture would be valuable information to explore more (especially through interviews as authors have suggested) in future research to find how to popularize kimono.

Is the work clearly and accurately presented and does it cite the current literature?

Partly

If applicable, is the statistical analysis and its interpretation appropriate?

I cannot comment. A qualified statistician is required.

Are all the source data underlying the results available to ensure full reproducibility?

Yes

Is the study design appropriate and is the work technically sound?

Yes

Are the conclusions drawn adequately supported by the results?

Partly

Are sufficient details of methods and analysis provided to allow replication by others?

Yes

Reviewer Expertise:

Female schoolteachers' dress code, society and culture, school curriculum

I confirm that I have read this submission and believe that I have an appropriate level of expertise to confirm that it is of an acceptable scientific standard.

## References

[1] : Impact Of Female School Teachers’ Dress Code on Job Performances: A Cross-Sectional Study in Saga City, Japan. *https://iojs.unida.ac.id/index.php/IJSR/article/view/62* .2020; 10.30997/ijsr.v2i3.62 10.30997/ijsr.v2i3.62

[2] : The issue of female teachers' clothing: one aspect of the modernization of women's clothing. *https://ndlsearch.ndl.go.jp/books/R000000004-I9773757* .2008;53: 10.18910/53380 31-44 10.18910/53380

# Response

**Published:** 2024-09-18

**Authors:** Kozue Miyashiro

**Affiliations:** Utsunomiya University, Utsunomiya, Tochigi Prefecture, Japan

We would like to sincerely thank you for taking the time to carefully review our manuscript and for providing such valuable feedback.

 Regarding your first suggestion, to distinguish between wearing kimono in everyday life and in the workplace, we have addressed this by adding the following footnote in the Introduction. we have also included citations for the two references you kindly recommended. Thank you again for your assistance.

 -------------------------------------------------------------

 It may be beneficial to distinguish between wearing kimono in daily life and wearing it in the workplace. Jayasooriya et al. (2020) conducted an interview-based study with Japanese female teachers, examining how the relaxation of dress codes in the workplace affected their professional performance. The findings suggest that while the duties of Japanese teachers are diverse, including not only teaching but also tasks such as supervising school-wide cleaning and assisting with school meals, the ability to wear casual attire facilitates their dynamic roles. Additionally, Namba (2008) argued that the transition from traditional Japanese clothing to Western-style attire in modern Japan has been obscured by preconceived notions. Namba's study investigates in detail the shifts in gender perspectives and aesthetic values, as well as the growing appreciation of the functionality of Western attire, which contributed to the change in clothing norms among female teachers.

 -------------------------------------------------------------

 Regarding your second suggestion—that the difference in age between the enthusiast group and the student group, in addition to the distinction of being an enthusiast or not, may introduce significant confounding factors—we have addressed this by incorporating the following revisions into the Conclusion as a limitation of this study. We believe this clarification has enhanced the paper by clearly outlining its limitations as well as its contributions. Thank you once again for your valuable insights.

 -------------------------------------------------------------

 The potential confounding between years of kimono enthusiasm and age also needs to be carefully treated: A significant disparity exists between the mean age of the college student group and that of the kimono enthusiasts, which may impact their daily routines and preferences for everyday attire. Moreover, age differences could influence their exposure to cultural values and knowledge, potentially leading to divergent attitudes towards kimono. Similarly, the finding that "the ease of movement in Western clothing becomes less significant as the experience of wearing kimono increases" must be carefully examined in future research to determine whether this applies more broadly to populations beyond kimono enthusiasts.

 -------------------------------------------------------------

# Reviewer report: Reviewer response for version 1

**DOI:** 10.5256/f1000research.163443.r270665

**Published:** 2024-05-22

**Authors:** Ellen McKinney

**Affiliations:** 1 The University of Alabama, Tuscaloosa, Alabama, USA

This study illuminates the occasions on which young people wear kimono and how they perceive the practice. It also compares this with the experiences of older people who regularly wear kimono.

 The literature review establishes the problem under investigation and serves as a foundation for this study. To provide a comprehensive context, it may be beneficial to incorporate the findings of previous related studies, including the one from which the survey questions are derived. These findings can then be compared and discussed at the conclusion of the paper, offering a more comprehensive understanding of the cultural significance of kimono wearing in Japan.

 The study method is robust and comprehensive. However, it is important to note that the statistical analysis is beyond the scope of my expertise, therefore, I am unable to provide detailed commentary on this aspect. 

 The conclusions and discussion provide good ideas for increasing students' interest in kimonos for daily wear and good ideas for future research, such as interviews.

 There are a few minor suggestions:

 The introduction should clearly list all hypotheses and research questions and serve as a reliable guide for our readers, ensuring a smooth navigation through the study.

 This link needs to be fixed. Please correct the following: Ministry of Economy, Trade and Industry in Japan, Textile Division: Report from the Secretariat (Questionnaire Survey Results).2015. (in Japanese). Retrieved March 2, 2024. Reference Source

Is the work clearly and accurately presented and does it cite the current literature?

Partly

If applicable, is the statistical analysis and its interpretation appropriate?

I cannot comment. A qualified statistician is required.

Are all the source data underlying the results available to ensure full reproducibility?

Yes

Is the study design appropriate and is the work technically sound?

Yes

Are the conclusions drawn adequately supported by the results?

Yes

Are sufficient details of methods and analysis provided to allow replication by others?

Yes

Reviewer Expertise:

Japanese fashion, Kimono, Zero-waste apparel design

I confirm that I have read this submission and believe that I have an appropriate level of expertise to confirm that it is of an acceptable scientific standard.

# Response

**Published:** 2024-06-18

**Authors:** Kozue Miyashiro

**Affiliations:** Utsunomiya University, Utsunomiya, Tochigi Prefecture, Japan

We apologize for the delayed response. We have received your review and sincerely appreciate the time and effort you have dedicated on our behalf.

# Response

**Published:** 2024-06-19

**Authors:** Kozue Miyashiro

**Affiliations:** Utsunomiya University, Utsunomiya, Tochigi Prefecture, Japan

In accordance with your suggestions, we have revised the paper to include a list of the hypotheses and research questions in the introduction and have submitted version 2 to the editor. Additionally, I apologize for the broken link in the references. I have updated the link to the correct website.
